# Clinical significance of L-type amino acid transporter 1 expression as a prognostic marker and potential of new targeting therapy in biliary tract cancer

**DOI:** 10.1186/1471-2407-13-482

**Published:** 2013-10-16

**Authors:** Kyoichi Kaira, Yutaka Sunose, Yasuhiro Ohshima, Noriko S Ishioka, Kazuhisa Arakawa, Tetsushi Ogawa, Noriaki Sunaga, Kimihiro Shimizu, Hideyuki Tominaga, Noboru Oriuchi, Hideaki Itoh, Shushi Nagamori, Yoshikatsu Kanai, Aiko Yamaguchi, Atsuki Segawa, Munenori Ide, Masatomo Mori, Tetsunari Oyama, Izumi Takeyoshi

**Affiliations:** 1 Department of Medicine and Molecular Science, Gunma University Graduate School of Medicine, Showa-machi, Maebashi, Gunma, Japan; 2 Department of Thoracic and Visceral Surgery, Gunma University Graduate School of Medicine, Showa-machi, Maebashi, Gunma, Japan; 3 Medical Radioisotope Application Group, Quantum Beam Science Directorate, Japan Atomic Energy Agency, Watanuki, 370-1292 Takasaki, Gunma, Japan; 4 Department of Surgery, Maebashi Red Cross Hospital, Asahi-cho, Maebashi, Gunma, Japan; 5 Department of Molecular Imaging, Gunma University Graduate School of Medicine, Showa-machi, Maebashi, Gunma, Japan; 6 Department of Diagnostic Radiology and Nuclear Medicine, Gunma University Graduate School of Medicine, Showa-machi, Maebashi, Gunma, Japan; 7 Department of Radiology, Saku Central Hospital, Usuda, Saku, Nagano, Japan; 8 Department of Pathology, Maebashi Red Cross Hospital, Asahi-cho, Maebashi, Gunma, Japan; 9 Division of Bio-system Pharmacology, Department of Pharmacology, Graduate School of Medicine, Osaka University, Osaka, Japan; 10 Department of Bioimaging Information Analysis, Gunma University Graduate School of Medicine, Showa-machi, Maebashi, Gunma, Japan; 11 Department of Diagnostic Pathology, Gunma University Graduate School of Medicine, Showa-machi, Maebashi, Gunma, Japan; 12 Oncology Center, Gunma University Hospital Showa-machi, 371-8511 Maebashi, Gunma, Japan

**Keywords:** LAT1, Biliary tract cancer, Amino acid transporter, Prognostic factor, BCH

## Abstract

**Background:**

The expression of L-type amino acid transporter 1 (LAT1) has been described to play essential roles in tumor cell growth and survival. However, it remains unclear about the clinicopathological significance of LAT1 expression in biliary tract cancer. This study was conducted to determine biological significance of LAT1 expression and investigate whether LAT1 could be a prognostic biomarker for biliary tract cancer.

**Methods:**

A total of 139 consecutive patients with resected pathologic stage I-IV biliary tract adenocarcinoma were retrospectively reviewed. Tumor specimens were stained by immunohistochemistry for LAT1, Ki-67, microvessel density determined by CD34, and p53; and prognosis of patients was correlated. Biological significance of LAT1 expression was investigated by *in vitro* and *in vivo* experiments with LAT inhibitor, 2-aminobicyclo-(2,2,1)-heptane-2-carboxylic acid (BCH) using cholangiocarcinoma cell line.

**Results:**

In total patients, high LAT1 expressions were recognized in 64.0%. The expression of LAT1 was closely correlated with lymphatic metastases, cell proliferation and angiogenesis, and was a significant indicator for predicting poor outcome after surgery. LAT1 expression was a significant independent predictor by multivariate analysis. Both *in vitro* and *in vivo* preliminary experiments indicated that BCH significantly suppressed growth of the tumor and yielded an additive therapeutic efficacy to gemcitabine and 5-FU.

**Conclusions:**

High expression of LAT1 is a promising pathological marker to predict the outcome in patients with biliary tract adenocarcinoma. Inhibition of LAT1 may be an effective targeted therapy for this distressing disease.

## Background

Biliary tract cancer is a relatively uncommon malignant neoplasm and is one of the aggressive malignancy with poor prognosis [1]. Gallbladder carcinoma and extrahepatic bile ducts carcinoma (cholangiocarcinoma) are the most common biliary tract cancer and cholangiocarcinoma is classified into intrahepatic and extrahepatic disease according to its anatomical location within the biliary tree [2]. Surgical resection remains the only potentially curative therapeutic option, however, more than half of patients present with unresectable disease. Even if curative resection can be performed, the 5-year overall survival is 20-32% for intrahepatic cholangiocarcinoma, 30-42% for hilar cholangiocarcinoma, and 18-54% for distal cholangiocarcinoma [3-5]. Although many patients may receive adjuvant chemotherapy to improve chance of cure, there is no established standard chemotherapy. In advanced biliary tract cancer, combination chemotherapy with gemcitabine and a platinum-based agent is regarded as a standard treatment, however, the prognosis after treatment remains dismal [6]. To date, the patients with biliary tract cancer lack a survival benefit if treated with chemotherapy or radiation therapy. Thus, we need a new effective therapy to improve the survival of patients. To improve the outcome of therapy, therefore, clinical markers that can predict response to the specific therapy and the prognosis should be established.

Amino acid transporters are essential for growth and proliferation of normal cells as well as transformed cells [7,8]. L-type amino acid transporter 1 (LAT1) is one of the L-type amino acid transporters, and transports large neutral amino acids such as leucine, isoleucine, valine, phenylalanine, tyrosine, tryptophan, methionine and histidine [9,10]. LAT1 requires covalent association with the heavy chain of 4F2 cell surface antigen (CD98) for its functional expression in plasma membrane [9]. LAT1 has been closely associated with cancerous or proliferative cells, and previous studies have shown LAT1 to be highly expressed in proliferating tissues, many tumor cell lines and primary human tumors [10-17]. In human tumor tissues, LAT1 expression has a close relationship with cell proliferation, angiogenesis and cell cycle regulator [18,19]. Recently, the expression of LAT1 has been described to be a significant factor indicating a poor outcome in various human cancers [12-17]. Moreover, the potential of targeting therapy for LAT1 had been suggested in tumor cell lines by the inhibition of LAT1 using 2-aminobicyclo-(2,2,1)-heptane-2-carboxylic acid (BCH) [20,21]. However, it remains unknown whether LAT1 expression has a clinical and pathological significance in patients with biliary tract cancer.

In the present study, we examined LAT1 expression in the resected tissue specimens to evaluate the clinicopathological and prognostic significance of LAT1 in patients with biliary tract cancer. LAT1 expression was correlated with pathological biomarkers such as cellular proliferation, cell cycle regulator (p53) and angiogenesis. In addition, *in vitro* and *in vivo* animal studies were performed to investigate the potential of LAT1 as a therapeutic biomarker in a novel targeting therapy.

## Methods

### Patients

We analyzed 157 consecutive patients with biliary tract adenocarcinoma who underwent surgical resection at Gunma University Hospital and Maebashi Red Cross Hospital between September 2000 and October 2011. Ten patients who received induction chemotherapy or radiation therapy were excluded. In all cases, magnetic resonance cholangiopancreatography (MRCP) and endoscopic retrograde cholangiopancreatography (ERCP) were performed before surgical resection, and pancreatic ductal adenocarcinoma and ampullary carcinoma were excluded from the study. The specimens from eight patients were not available. All surgical specimens were reviewed and classified according to the WHO classification by an experienced pathologist who was unaware of clinical or imaging findings. Patients with pathological diagnosis other than adenocarcinoma were excluded. In total, 139 patients were analyzed in the study. The study population consisted of patients with extrahepatic cholangiocarcinoma (EHCC), intrahepatic cholangiocarcinoma (IHCC) and gallbladder carcinoma (GB). Pathologic tumor-node-metastasis (TNM) stages were established using the International System for Staging bile duct cancer adopted by the American Joint Committee on Cancer and the Union Internationale Centre le Cancer [22].

We also analyzed a control group of 16 patients with surgically resected benign biliary tract lesions. Immunohistochemical staining of samples from these 16 patients was performed and compared with that of biliary tract cancer. The pathological diagnosis of the control group was as follows: 6 patients with cholesterol polyp, 4 patients with hyperplastic polyp, 3 patients with xanthogranulomatous chlecystitis and 3 patients with adenomyomatosis. This study was approved by the institutional review board of Gunma University Hospital (ethical committee for clinical studies-Gunma University faculty of Medicine) and written informed consent was obtained from all of the patients or their families who participated to this study.

### Immunohistochemical staining

LAT1 expression was determined by immunohistochemical staining with LAT1 antibody (2 mg/mL, anti-human monoclonal mouse antibody, 4A2, provided by Dr H. Endou [J-Pharma, Tokyo, Japan], dilution; 1:3200). The production and characterization of the LAT1 antibody has previously been described [15]. The detailed protocol for immunostaining was published elsewhere [16]. The LAT1 expression score was assessed by the extent of staining as follows: 1, ≤ 10% of tumor area stained; 2, 11-25% stained; 3, 26-50% stained; and 4, ≥51% stained. The tumors in which stained tumor cells were scored as 3 or 4 were defined as high expression.

For CD34, Ki-67 and p53, immunohistochemical staining was performed according to the procedures described in previous reports [23,24]. The following antibodies were used: mouse monoclonal antibodies against CD34 (Nichirei, Tokyo, Japan, 1:800 dilution), Ki-67 (Dako, Glostrup, Denmark, 1:40 dilution), and p53 (D07; Dako, 1:50 dilution). The number of CD34-positive vessels was counted in four selected hot spots in a x 400 field (0.26 mm^2^ field area). Microvessel density (MVD) was defined as the mean count of microvessels per 0.26 mm^2^ field area. The median number of CD34-positive vessels was evaluated, and the tumors in which stained tumor cells made up more than each median value were defined as high expression. For p53, microscopic examination for the nuclear reaction product was performed and scored, and p53 expression in greater than 10% of tumor cells was defined as positive expression [24]. For, Ki-67, a highly cellular area of the immunostained sections was evaluated. All epithelial cells with nuclear staining of any intensity were defined as high expression. Approximately 1000 nuclei were counted on each slide. Proliferative activity was assessed as the percentage of Ki-67-stained nuclei (Ki-67 labeling index) in the sample. The median value of the Ki-67 labeling index was evaluated, and the tumor cells with greater than the median value were defined as high expression. The sections were assessed using a light microscopy in a blinded fashion by at least two of the authors.

### Biochemical materials

Dulbecco’s modified Eagle’s medium (DMEM), penicillin and streptomycin were purchased from WAKO Pure Chemical Industries (Osaka, Japan). BCH was obtained from NARD Institute (Hyogo, Japan). 3-[4,5-dimethyl-2-thiazolyl]-2,5-diphenyl-*2H*-tetrazolium bromide (MTT) were purchased from Dojindo Laboratories (Kumamoto, Japan). All other chemicals used were of the highest purity available.

### Cell culture

A human cholangiocarcinoma cell lines, HuCCT1 (JCRB0425), OZ (JCRB1032), and HuH28 (JCRB0426) were purchased from the Health Science Research Resources Bank (Osaka, Japan) [25-27], and routinely maintained in DMEM containing 10% heat-inactivated fetal bovine serum (AusGeneX, Loganholme, QLD, Australia), penicillin (100 units/ml), streptomycin (100 μg/ml) and L-glutamine (2 mM) at 37°C in 5% CO_2_, 95% air.

### Expression of LAT mRNA in cholangiocarcinoma

Previously, 4 subtypes of L-type amino acid transporter (LAT1-4) have been identified [8,23-30]. Realtime RT-PCR analysis was performed to determine the expression of LAT1, LAT2, LAT3, and LAT4 mRNA in cholangiocarcinoma cell line. Total RNA was isolated from HuCCT1 cells using a Fast Pure RNA kit (Takara Bio, Shiga, Japan). The first-strand complement DNA was synthesized from 0.5 μg of total RNA with PrimeScript Reverse Transcriptase (Takara Bio). The sequences of specific primers were shown in Additional file 1: Table S1 (online only). The realtime PCR analysis was performed by first incubating each complement DNA sample with the primers (0.5 μM each) and Thunderbird SYBR qPCR Mix (Toyobo, Osaka, Japan). Amplification was carried out for 40 cycles (95°C for 15 s, 60°C for 30 s) with Piko-Real thermal cycler (Thermo Fisher Scientific, Waltham, MA). The data was analyzed according to 2^-ΔΔC^_T_ method (internal control: β-actin, calibrator: LAT1).

### Suppression of cell proliferation with LAT1 inhibition

Cells were plated at a concentration of 1 x 10^3^ cells/well in 96-well plates and incubated in the growth medium for 24 h. At first, in order to determine the effect of LAT1 inhibition on cholangiocarcinoma, HuCCT1 cells were treated with BCH (0.1, 1, 2, 3, 5, 10, 20, 30, or 100 mM) and incubated for 6 days. Next, the effect of LAT1 inhibition on the antitumor activity of gemcitabine (GEM, Eli Lilly, Indianapolis, IN) or 5-fluorouracil (5-FU, Kyowa Hakko Kirin, Shizuoka, Japan) was evaluated. Cells were incubated for 6 days with GEM (10, 20, 50 or 100 nM) or 5-FU (1, 10, or 100 μM) in a presence or absence of 10 mM BCH. Then, cells were incubated with 0.5 mg/ml MTT for 4 h at 37°C. The resulting formazan was solubilized, and the absorbance was read at 590 nm with a microtiter plate reader (Vmax; Molecular Devices, Sunnyvale, CA).

### Suppression of amino acid uptake into cells with LAT1 inhibition

Inhibition of amino acid transport by BCH was examined using [^14^C]L-leucine (Perkin-Elmer Life Sciences, Boston, MA), one of the substrates of LATs [31]. HuCCT1 cells (1.0 x 10^5^ cells/well) were plated in the 24-well plates and incubated in the growth medium for 24 h. After the incubation, the cells were washed three times with sodium-free Hunk’s balanced salt solution (Na^+^-free HBSS; 137 mM choline chloride, 5.3 mM KC1, 1.3 mM CaCl_2_, 0.49 mM MgCl_2_, 0.41 mM MgSO_4_, 0.35 mM K_2_HPO_4_, 0.44 mM KH_2_PO_4_, 4.2 mM KHCO_3_, 5.6 mM D-glucose (pH 7.4)). The cells were incubated in Na^+^-free HBSS containing various concentration of BCH (0.01, 0.03, 0.1, 0.3, 1, or 3 mM) for 10 min at 37°C, and then, the supernatant was replaced by Na^+^-free HBSS containing 1 μM [^14^C]L-leucine and BCH with the same concentration (0.01, 0.03, 0.1, 0.3, 1, or 3 mM). At 1 min after treatment with [^14^C]L-leucine, uptake was terminated by removing the uptake solution followed by washing three times with ice-cold Na^+^-free HBSS. Cells were solubilized with 0.1 N NaOH, and radioactivity was measured by liquid scintillation spectrometry (AccuFLEX LSC-7200, Hitachi Aloka Medical, Tokyo, Japan).

### Immunoblotting

Cells were dissolved in sample buffer (25% glycerin, 1% SDS, 62.5 mM Tris-Cl, 10 mM dithiothreitol) and incubated at 65°C (LAT1) or 95°C (CD98 and β-actin) for 15 min. Aliquots of samples containing 40 μg of protein were analyzed by 10% SDS-polyacrylamide gel electrophoresis and transferred onto a polyvinylidene difluoride membrane. Blots were incubated at 4°C overnight in 10 mM Tris–HCl, 100 mM NaCl, 0.1% Tween 20, pH 7.5 (TBST), with 5% skim milk and then with rabbit anti-LAT1 C-terminus antibody (1:5,000) [32], rabbit anti-CD98 antibody (1:200; Santa Cruz Biotechnology) or rabbit anti-actin antibody (1:1,000; Cell Signaling Technology, Beverly, MA) at 4°C overnight. After having been washed with TBST, the blots were incubated with goat horseradish peroxidase conjugated anti-rabbit IgG antibody (1:20,000; Cell Signaling Technology) for 1.5 h at room temperature. The blots were further washed with TBST, and specific proteins were visualized by using enhanced chemiluminescence western blotting detection reagents (GE Healthcare, Piscataway, NJ).

### Anti-tumor effect of LAT1 inhibition

Five-week-old male BALB⁄ c nude mice were purchased from CLEA Japan (Tokyo, Japan). The animals were cared for and treated in accordance with the guidelines of the animal care and experimentation committee at our facility. HuCCT1 cells (1 x 10^7^ cells) were inoculated s.c. into the flank of the mice. After inoculation, the longer and shorter diameters of the tumor were measured with caliper and tumor volume was calculated by the following formula: Tumor volume (mm^3^) = longer diameter x (shorter diameter)^2^ / 2. After tumor volumes had reached approximately 100 mm^3^, the mice were divided into control group and treatment group (n = 10). Saline or BCH (200 mg/kg) was intravenously administered once daily from the day of grouping (day 0) for 14 days. Tumor volume and body weight were measured two or three times a week for 42 days. No animals were excluded and no animals died due to toxicity.

To evaluate the effect of BCH on the tumor glucose metabolism, positron emission tomography (PET) imaging of tumor-bearing mice was performed with [^18^F]fluoro-2-deoxyglucose (^18^F-FDG) using an animal PET scanner (Inveon, Siemens, Knoxville, TN). ^18^F was produced using a cyclotron (CYPRIS HM-18, Sumitomo Heavy Industries, Tokyo, Japan) and ^18^F-FDG was synthesized in our facility. Mice for PET imaging were randomly selected from treatment group and control. Before imaging, mice were fasted for 8 h and had free access to water. ^18^F-FDG (10 MBq) was administered intravenously into mice followed by 10 min data acquisition at 2 h after the administration. Mice were maintained under isoflurane anesthesia during the administration, uptake period and PET scan. For analysis of the image, region of interest (ROI) was drawn around the edge of the tumor activity using ASIPro VM (CTI Concorde Microsystems, Knoxville, TN). The maximum and median activities were recorded. Standardized uptake value (SUV) was used to evaluate glucose metabolism of the tumor. SUV was calculated as follows: SUV = ROI activity (kBq/ml) / injected dose (MBq) x body weight (kg). SUV max and SUV 50% were compared between BCH-treated mice and control mice.

### Statistical analysis

Probability values of <0.05 indicated a statistically significant difference. Results are expressed as mean ± SEM. The significance of difference was determined by Student’s t-test. The correlation between different variables was analyzed using the nonparametric Spearman’s rank test. The Kaplan-Meier method was used to estimate survival as a function of time, and survival differences were analyzed by the log-rank test. Overall survival (OS) was determined as the time from tumor resection to death from any cause. Progression-free survival (PFS) was defined as the time between tumor resection and the first disease progression or death. Multivariate analyses were performed using stepwise Cox proportional hazards model to identify independent prognostic factors. Statistical analysis was performed using GraphPad Prism 4 software (Graph Pad Software, San Diego, CA, USA) and JMP 8 (SAS, Institute Inc., Cary, NC, USA) for Windows.

## Results

### Patient’s demographics

One hundred thirty-nine patients with biliary tract adenocarcinoma were analyzed (EHCC, n = 89; GB, n = 30; and IHCC, n = 20). Clinicopathologic results stratified by tumor location are listed in Table 1. The age of the patients ranged from 42 to 86 years, and the median age was 71 years. Most tumors (n = 126, 90.6%) were pathological stages I to III. Fifty-one patients had received postoperative adjuvant chemotherapy with GEM, S-1 (Taiho Pharmaceutical Co., Ltd, Tokyo, Japan) or oral administration of tegafur (a fluorouracil derivative drug). Intraoperative therapy was not performed on any patients. The day of surgery was considered the starting day for measuring postoperative survival. A median follow-up duration for all patients was 18.6 months (range, 3.0 to 110.3 months).

**Table 1:** Patient’s characteristics and pathological findings

**Characteristic**	**All patients (n = 139)**		**EHCC (n = 89)**		**GB (n = 30)**		**IHCC (n = 20)**		**Control (n = 16)**	
	**No. of patients**	**%**	**No. of patients**	**%**	**No. of patients**	**%**	**No. of patients**	**%**	**No. of patients**	**%**
**Median age (years)**	71		71		74		64		62	
**Male**	86	61.8	65	73.0	8	26.7	13	65.0	NA	
**R0 resection**	67	48.2	38	42.7	19	47.4	10	50.0	NA	
**Poorly differentiated**	33	23.7	18	20.2	3	10.0	12	60.0	NA	
**Lymphatic permeation**	111	79.8	74	83.1	23	76.7	14	70.0	NA	
**Vascular invasion**	93	66.9	66	74.1	16	53.3	12	60.0	NA	
**Lymph node metastases**	62	44.6	41	46.1	11	36.7	10	50.0	NA	
**UICC p-stage**										
**1**	40	28.7	23	25.8	12	40.0	5	25.0	NA	
**2**	70	50.4	49	55.1	12	40.0	9	45.0		
**3**	16	11.5	8	9.0	5	16.7	3	15.0		
**4**	13	9.4	9	10.1	1	3.3	3	15.0		
**Papillary morphology**	32	23.0	13	14.6	18	60.0	1	5.0	NA	
**Adjuvant chemotherapy**	51	36.7	34	38.2	8	26.7	9	45.0	NA	
**CEA (high)**	65	46.7	46	51.7	12	40.0	7	35.0	NA	
**CA19-9 (high)**	66	47.5	44	49.4	12	40.0	10	50.0	NA	
**LAT1 (high)**	89	64.0	59	66.3	18	60.0	12	60.0	0	0.0%
**Ki-67 (high)**	62	44.6	43	48.3	13	43.3	6	30.0	0	0.0%
**CD34 (high)**	69	49.6	46	51.7	14	46.7	9	45.0	0	0.0%
**p53 (positive)**	71	51.1	44	49.4	20	66.7	7	35.0	0	0.0%

*Abbreviation*: *EHCC* Extrahepatic cholangiocarcinoma, *GB* Gallbladder carcinoma, *IHCC* Intrahepatic cholangiocarcinoma, *UICC* International union against cancer, p-stage Pathological stage, *CEA* Carcinoembryonic antigen, *LAT1* L-type amino acid transporter 1, *NA* Not applicable.

### Immunohistochemical analysis

**Figure 1:**
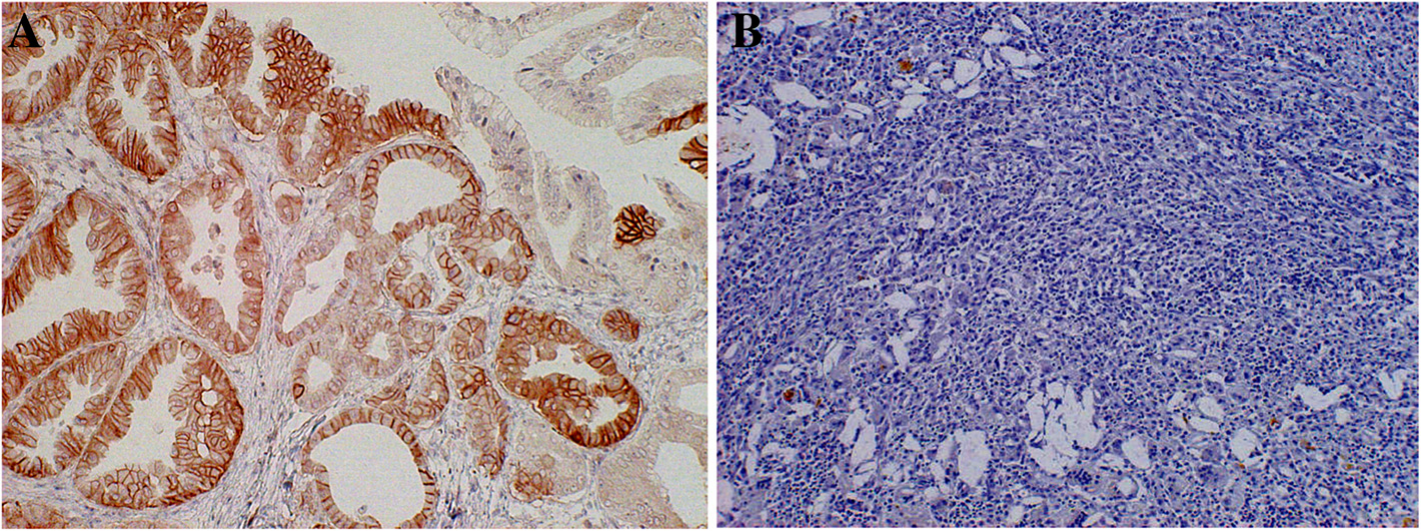
**Immunohistochemical staining of tissue from a 79-years old man with extrahepatic cholangiocarcinoma (A) and a 66-years old woman with Xanthogranulomatous chlecystitis as control group (B).** Immunostaining of LAT1 demonstrates a membranous immunostaining pattern in cholangiocarcinoma, but there was no evidence of LAT1 staining in xanthogranulomatous chlecystitis.

The immunohistochemical analysis was performed on the 139 primary lesions with cholangiocarcinoma and 16 resected lesions with biliary benign diseases. Figure 1 represents the immunohistochemical staining of LAT1 expression. LAT1 immunostaining was detected in carcinoma cells in tumor tissues and localized predominantly on their plasma membrane. All positive cells revealed strong membranous LAT1 immunostaining. Cytoplasmic staining was rarely evident. The high expression rate and average scoring of LAT1 were compared according to tumor location (Additional file 2: Table S2, online only). In total patients, the high expression rate and average scoring of LAT1 were recognized in 64.0% and 2.7 ± 0.9, respectively.

Based on the results of analysis on cholangiocarcinoma, cutoff points for high CD34 expression and high Ki-67 labeling index were defined as follows. The median number of CD34-positive vessels was 21 (range, 4–52), and the value of 21 was chosen as a cutoff point. The median value of the Ki-67 labeling index was 35% (range, 2–76), and the value of 35% was chosen as cutoff point. Positive expression of p53 was recognized in 51.1% (71/139). Table 1 shows the expression status of these biomarkers according to tumor location. Rate of high expression or positivity in these biomarkers was significantly higher in cholangiocarcinoma than in biliary benign lesions (Table 1). Patient’s demographics according to LAT1 expression status are listed in Table 2. The expression of LAT1 was significantly associated with lymphatic permeation, vascular invasion, lymph node metastasis, CA19-9, Ki-67, and MVD.

**Table 2:** Patient’s demographics according to LAT1 expression status

**Parameter**		**All patient (n = 139)**			**Extrahepatic CC (n = 89)**			**Gallbladder carcinoma (n = 30)**			**Intrahepatic CC (n = 20)**		
		**High(n = 89)**	**Low (n = 50)**	***p*-value**	**High (n = 59)**	**Low (n = 30)**	***p*-value**	**High (n = 18)**	**Low (n = 12)**	***p*-value**	**High (n = 12)**	**Low (n = 8)**	***p*-value**
Age	≤65 / >65	24 / 65	11 / 39	0.549	17 / 42	4 / 26	0.121	3 / 15	2 / 10	>0.999	5 / 7	5 / 3	0.649
Gender	M / F	55 / 34	31 / 19	>0.999	43 / 16	22 / 8	>0.999	4 / 14	4 / 8	0.677	8 / 4	5 / 3	>0.999
Tumor size(mm)	≤35 / >35	48 / 41	28 / 22	0.862	34 / 25	22 / 8	0.170	11 / 7	10 / 2	0.248	4 / 8	4 / 4	0.647
Resection status	R0 / R1	42 / 47	25 / 25	0.859	25 / 34	13 / 17	>0.999	12 / 6	8 / 4	>0.999	5 / 7	5 / 3	0.649
Pathological differentiation	WD or MD / PD	67 / 22	39 / 11	0.572	46 / 13	25 / 5	0.780	16 / 2	11 / 1	0.377	5 / 7	3 / 5	>0.999
Lymphatic permeation	Yes / No	78 / 11	33 / 17	**0.003**	53 / 6	9 / 21	**<0.001**	16 / 2	7 / 5	0.459	9 / 3	5 / 3	0.642
Vascular invasion	Yes / No	68 / 21	25 / 25	**0.002**	49 / 10	17 / 13	**0.011**	11 / 7	4 / 8	0.263	8 / 4	4 / 4	0.647
Lymph node metastasis	Yes / No	51 / 38	11 / 39	**<0.001**	33 / 26	8 / 22	**0.013**	9 / 9	2 / 10	0.121	9 / 3	1 / 7	**0.019**
Disease staging	I or II / III or IV	64 / 25	45 / 5	0.098	43 / 16	27 / 3	0.099	12 / 6	12 / 0	0.056	9 / 3	6 / 2	>0.999
Papillary pattern	Yes / No	18 / 71	14 / 36	0.302	8 / 51	5 / 25	0.755	10 / 8	8 / 4	0.708	0 / 12	1 / 7	0.400
Adjuvant chemotherapy	Yes / No	40 / 49	11 / 39	**0.009**	28 / 31	6 / 24	**0.012**	7 / 11	1 / 11	0.099	5 / 7	4 / 4	>0.999
CEA	≤2.1 / >2.1	45 / 44	29 / 21	0.479	26 / 33	13 / 17	>0.999	11 / 7	7 / 5	>0.999	8 / 4	5 / 3	>0.999
CA19-9	≤45.1 / >45.1	32 / 57	37 / 13	**<0.001**	22 / 37	23 / 7	**<0.001**	9 / 9	9 / 3	0.259	5 / 7	5 / 3	0.649
Ki-67	High / Low	47 / 42	15 / 35	**0.012**	33 / 26	10 / 20	0.072	10 / 8	3 / 9	0.141	4 / 8	2 / 6	>0.999
p53	P / N	48 / 41	23 / 27	0.383	32 / 27	12 / 18	0.263	12 / 6	8 / 4	>0.999	4 / 8	3 / 5	>0.999
CD34	High / Low	51 / 38	18 / 32	**0.021**	34 / 25	12 / 18	0.124	11 / 7	3 / 9	0.071	6 / 6	3 / 5	0.669

*Abbreviation*: *LAT1* L-type amino acid transporter 1, *CC* Cholangiocarcinoma, *M / F* Male / Female, *CEA* Carcinoembryonic antigen, *WD or MD / PD* Well differentiated or moderate differentiated / poorly differentiated, *P /N* Positive / Negative, *Bold numbers* Statistically significant difference.

### Correlation between LAT1 expression and other biomarkers

Analysis with Spearman’s rank correlation revealed that LAT1 expression was significantly correlated with Ki-67 and CD34 in all tumor location except CD34 in IHCC (Additional file 3: Table S3, online only).

### Univariate and multivariate survival analysis

**Figure 2:**
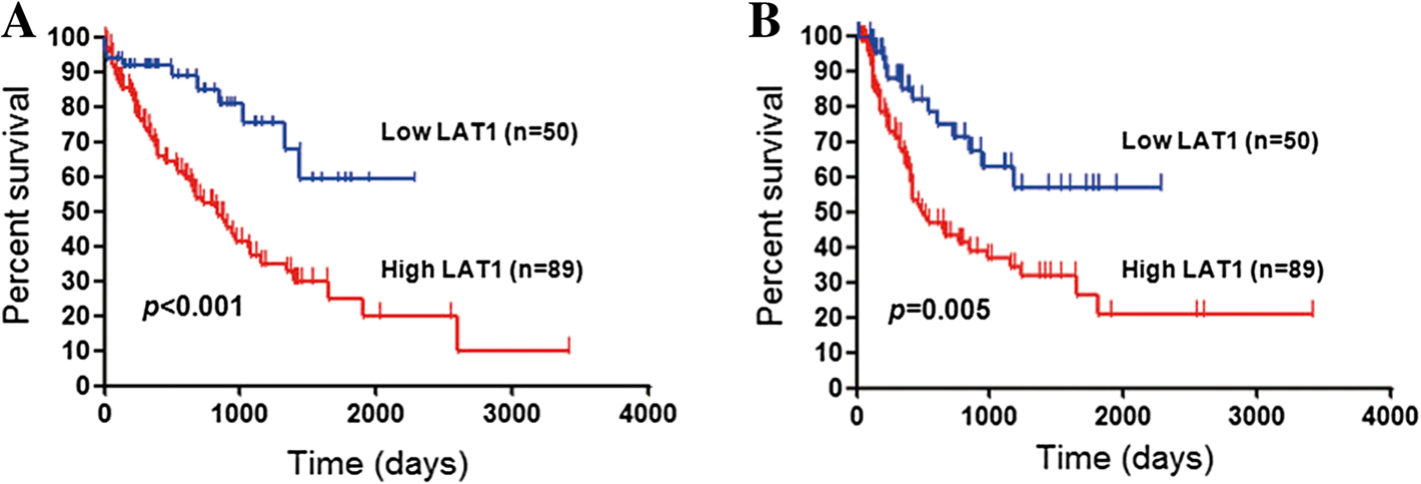
**Outcomes after surgical resection shown by Kaplan-Meier analysis of overall survival (OS) and progression-free survival (PFS) according to LAT1 and CD98 expression.** A statistically significant difference in OS **(A)** and PFS **(B)** was observed between patients with high and low LAT1 expression.

In all patients, the 5-year survival rate and median survival time (MST) for OS were 35.6% and 1073 days, respectively, and the 3-year survival rate and MST for PFS was 45.1% and 840 days, respectively. Because of a postoperative recurrence, 39 patients received systemic chemotherapy using GEM or S-1. Table 3 shows the univariate and multivariate analysis in all patients (n = 139). Univariate analysis revealed that significant variables for OS were resected status, tumor differentiation, lymphatic permeation, vascular invasion, lymph nodes metastasis, LAT1, and Ki-67. Significant prognostic markers for PFS by the univariate analysis included resected status, tumor differentiation, lymphatic permeation, vascular invasion, lymph node metastasis, tumor stage, and LAT1. According to the results of univariate log-rank test, we screened prognostic factors with cut-off of *p* < 0.05. Multivariate analysis confirmed that lymphatic permeation and a high LAT1 expression, lymphatic permeation and Ki-67 were independent prognostic factors for predicting poor OS, and lymphatic permeation and vascular invasion for poor PFS. Figure 2 shows the Kaplan-Meier survival curve in patients with high and low for LAT1 expression.

**Table 3:** Univariate and multivariate analysis in overall survival and progression-free survival

**Variable**	**Overall survival**					**Progression-free survival**				
	**5-year survival rate (%)**	***p*-value (univariate)**	***p*-value (multivariate)**	**Hazard ratio**	**95% CI**	**3-year survival rate (%)**	***p*-value (univariate)**	***p*-value (multivariate)**	**Hazard ratio**	**95% CI**
**Anatomical locations**										
EHCC	38.1	0.837				48.3	0.395			
IHCC	28.0					28.2				
GB	34.5					45.6				
**Age**										
≤65 yr	39.9	0.095				48.4	0.707			
≻65 yr	27.5					54.7				
**Gender**										
Male	30.2	0.267				49.7	0.634			
Female	33.5					58.6				
**Resection**					0.974 to 1.752					0.875 to 1.535
R0	42.5	**0.026**	0.075	1.300		64.2	**0.016**	0.310	1.154	
R1 or R2	29.8					40.1				
**Tumor differentiation**					0.881 to 1.593					0.729 to 1.307
Well or moderate	47.3	**0.017**	0.251	0.190		55.2	**0.008**	0.845	0.971	
Poorly	7.8					39.6				
**Lymphatic permeation**					1.057 to 7.629					1.212 to 10.72
Yes	19.9	**0.002**	**0.036**	2.555		44.9	**<0.001**	**0.016**	3.139	
No	79.9					81.7				
**Vascular invasion**				0.939	0.468 to 1.939					1.073 to 5.057
Yes	20.5	**0.011**	0.862			40.2	**<0.001**	**0.031**	2.244	
No	58.7					74.8				
**Lymph nodes metastasis**				0.977	0.552 to 1.706					0.517 to 4.534
Positive	20.5	**0.041**	0.935			41.3	**0.003**	0.454	1.507	
Negative	44.0					63.2				
**Tumor stage**										0.922 to 2.980
I or II	38.2	0.051				60.1	**0.003**	0.088	1.685	
III or IV	29.5					26.8				
**LAT1**					1.196 to 5.321					0.785 to 2.837
High	20.3	**<0.001**	**0.013**	2.414		41.1	**0.005**	0.242	1.449	
Low	59.3					71.3				
**Ki-67**					1.030 to 3.093					
High	29.6	**0.021**	**0.038**	1.781		49.2	0.192			
Low	36.2					53.6				
**p53**										
Positive	21.3	0.119				54.1	0.831			
Negative	38.1					51.6				
**CD34**										
High	28.6	0.349				51.1	0.696			
Low	42.8					52.2				

*Abbreviation*: *95% CI* 95% confidence interval, *EHCC* Extrahepatic cholangiocarcinoma, *IHCC* Intrahepatic cholangiocarcinoma, *GB* Gallbladder carcinoma, *CEA* Carcinoembryonic antigen, *LAT1* L-type amino acid transporter 1, *Bold numbers* Statistically significant difference.

### Expression of LAT1 and CD98 in human cholangiocarcinoma cell lines

As shown in Additional file 4: Figure S1 (online only), both LAT1 and CD98 were expressed in all three human cholangiocarcinoma cell lines, HuCCT1, OZ, and HuH28. The expression level of LAT1 in OZ was lower than that of the other cell lines. HuCCT1 cell was used in the following experiments because of its higher expression of LAT1 and tumorigenesis in nude mice.

### LAT inhibition suppresses cellular amino acid transport and proliferation through LAT1

**Figure 3:**
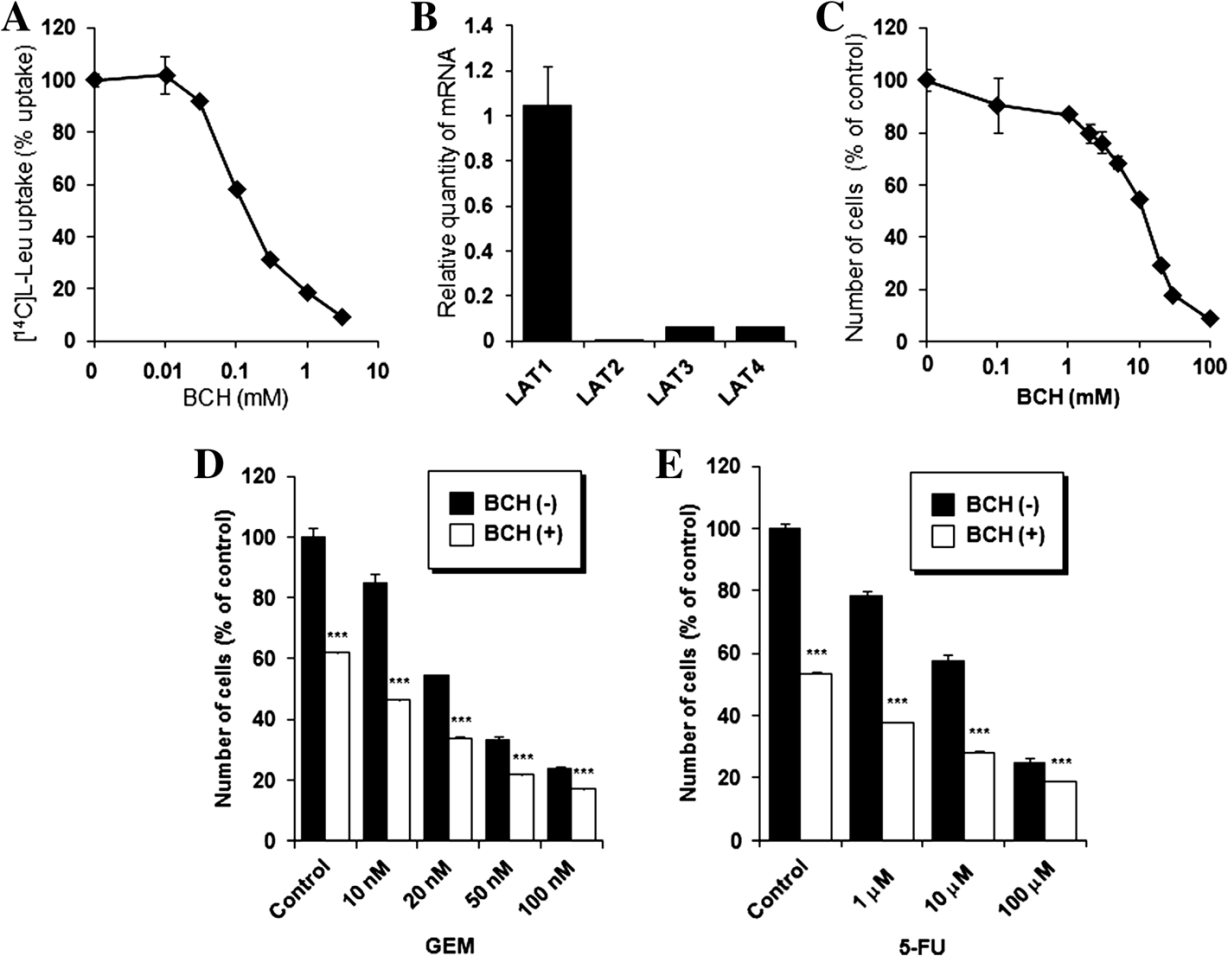
**Effect of LAT inhibition on *in vitro *cellular proliferation and anti-tumor activity of GEM and 5-FU: (A)** BCH inhibits [^14^C]L-leucine uptake concentration-dependently in HuCCT1 cells (n = 4). Ordinate shows a percentage of [^14^C]L-leucine uptake in the absence of BCH as a control. **(B)** Expression of LAT1, LAT2, LAT3, and LAT4 mRNA in HuCCT1 cells (n = 4). Ordinate shows relative quantity of mRNA calibrated by LAT1 mRNA. **(C)** BCH decreases number of HuCCT1 cells concentration-dependently (n = 4). Ordinate shows number of cells in a percentage of control (without BCH). Addition of 10 mM BCH enhances anti-tumor effect of GEM **(D)** and 5-FU **(E)** on HuCCT1 cells. Ordinate shows number of cells in a percentage of control (n = 4). A statistically significant difference from the control is indicated by *** (P < 0.001).

The cellular uptake of [^14^C]L-leucine was measured in a presence of various concentrations of BCH, and was inhibited concentration-dependently by the treatment with BCH (Figure 3A). Expression profile of LAT1-4 in HuCCT1 examined by realtime RT-PCR showed that the expression of LAT1 was extremely higher than the other LATs (Figure 3B). These results indicate that BCH inhibits amino acid transport through LAT1 in HuCCT1 cells. Furthermore, BCH decreased number of cells concentration-dependently (Figure 3C), indicating that BCH could inhibit proliferation of HuCCT1 cells through inhibition of amino acid uptake.

### LAT inhibition enhances anti-tumor activity of GEM and 5-FU

As shown in Figure 3D and E, combination of BCH with chemotherapeutic agents decreased number of HuCCT1 cells. Cytotoxicity of GEM and 5-FU was significantly enhanced in combination with 10 mM BCH, indicating additive effect of LAT inhibitor on anti-tumor activity of GEM and 5-FU in HuCCT1.

### LAT inhibition suppresses growth of xenografts in nude mice

**Figure 4:**
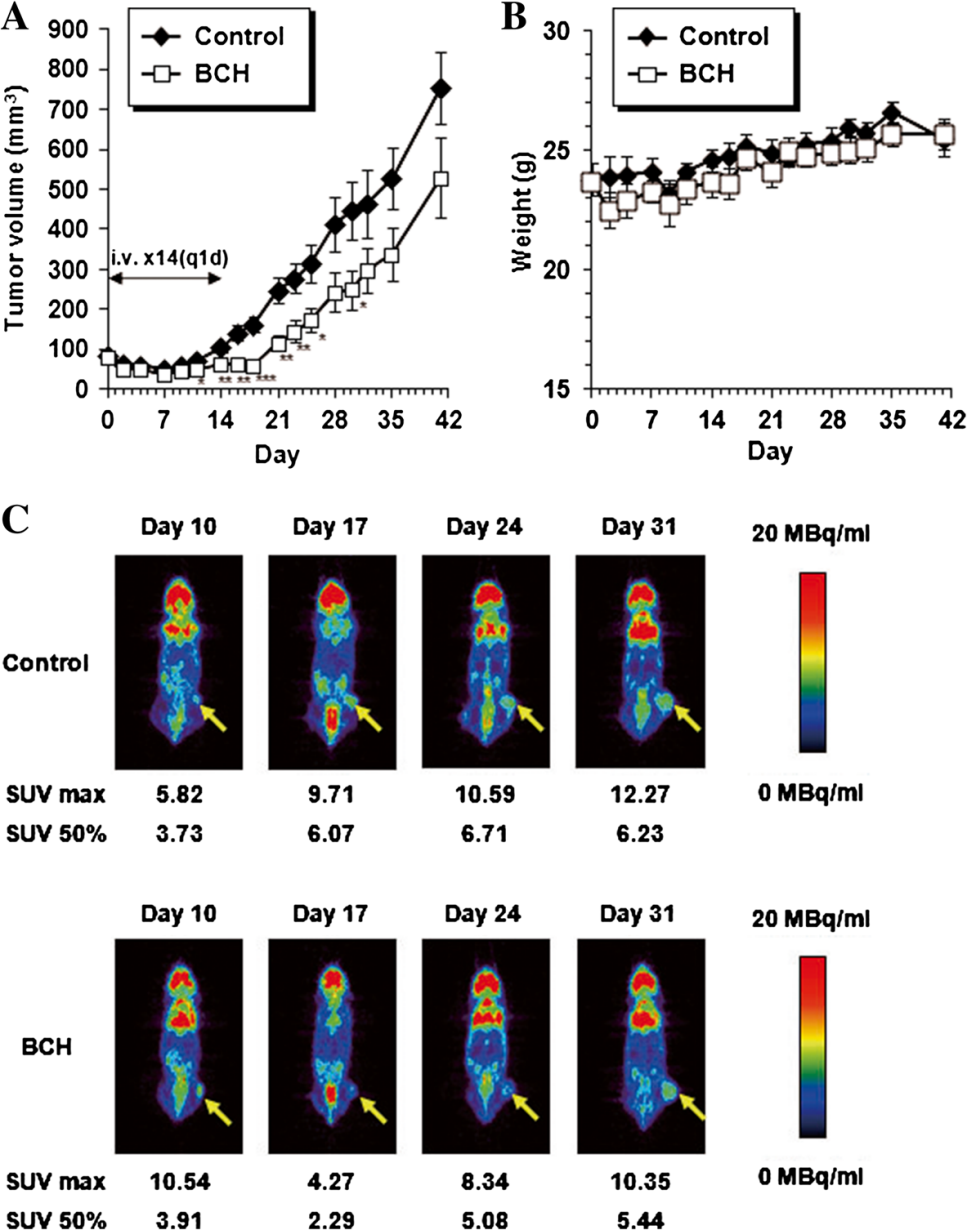
***In vivo *anti-tumor effect of LAT inhibition on cholangiocarcinoma xenograft. (A)** Intravenous administration of BCH shows delay in the growth of HuCCT1 tumor (n = 10). A statistically significant difference from the control is indicated by * (P < 0.05), ** (P < 0.01), and *** (P < 0.001). **(B)** Changes in the body weight of HuCCT1 tumor-bearing mice after administration of BCH (n = 10). **(C)** Representative coronal section of ^18^F-FDG PET images of HuCCT1-bearing mice at 2 h after ^18^F-FDG injection. PET imaging was performed at indicated day after the day of grouping (n = 2). The calibration bar is shown at right-side of images. SUV max and SUV 50% are shown below the images.

Anti-tumor activity of BCH on cholangiocarcinoma was examined *in vivo* using HuCCT1-bearing mice. Daily administration of BCH (200 mg/kg) for 14 days caused statistically significant delay in the tumor growth up to 3 weeks after the completion of dosing (Figure 4A). There was no change in the body weight by the treatment with BCH (Figure 4B). Anti-tumor effect of BCH was also monitored using ^18^F-FDG PET to determine the decrease in the metabolism of the tumor. SUV max and SUV 50% of ^18^F-FDG were decreased at day 17 and increased thereafter in BCH-treated mice (Figure 4C).

## Discussion

This is the first study to elucidate the clinicopathologic significance of LAT1 expression in patients with biliary tract cancer. The expression of LAT1 in the tumor specimens was closely correlated with lymphatic metastases, cell proliferation, and angiogenesis; and was a significant indicator for predicting poor outcome after surgical resection. Therefore, a high LAT1 expression may play an important role on the growth of biliary tract cancer. No anatomic site-related differences were observed for LAT1. Results of our preliminary experiments indicated that the inhibition of LAT1 had significant anti-tumor effect on cholangiocarcinoma with acceptable toxicity and yielded an additive therapeutic efficacy to GEM and 5-FU. Our data suggests that LAT1 inhibition suppresses the growth of biliary tract cancer and LAT1 could be a potential target for locally advanced or metastatic biliary tract cancer.

Recently, two studies have exhibited the significance of LAT1 expression as a prognostic predictor in pancreatic cancer [33,34]. In pancreatic cancer, LAT1 was highly expressed in 52.6% [33]. In biliary tract cancer, the ratio of high LAT1 expression yielded a similar tendency among all anatomic site (EHCC, IHCC, and GB). These results indicate that the expression of LAT1 is higher in biliary tract cancer than pancreatic cancer. The LAT1 expression is variable in human cancers, and relatively low in adenocarcinoma, for example, 29% in pulmonary adenocarcinoma [12], 22% in prostate cancer [15], 43% in breast cancer [17], and 43% in gastric cancer [16]. LAT1 seemed to be expressed at higher level in biliary tract adenocarcinoma than in adenocarcinoma of the other organs. Therefore, LAT1 may play a crucial role in enhancing the cell proliferation and tumor growth in biliary tract cancer.

Recently, we had evaluated the protein expression of LAT1 by immunohistochemistry in patients with pulmonary neuroendocrine tumors [35]. Our data indicated that the expression of LAT1 tended to increase from low-grade to high-grade malignancies. Moreover, we confirmed the different expression of LAT1 between pancreatic cancer and pancreatic adenoma, showing that LAT1 expression was not observed in pancreatic adenoma, whereas LAT1 was highly expressed in pancreatic cancer [33]. Previous experimental data also demonstrated that LAT1 is overexpressed in tumor cells and LAT2 is dominantly expressed in normal cells [9,10]. In the protein expression level of human tissue specimens, there was no evidence of LAT1 expression in normal tissues. Thus, we believe that LAT1 is tumor-specific amino acid transporter and has a potential target of cancer therapeutics.

This study investigated the therapeutic potential of LAT1 inhibition in cholangiocarcinoma. We found that BCH as a competitive LAT inhibitor suppressed proliferation of cholangiocarcinoma cells and yielded an additive therapeutic efficacy to GEM and 5-FU *in vitro*. Moreover, *in vivo* experiment demonstrated significant growth suppression of tumor with acceptable toxicity. Recent reports also showed that the inhibition of LAT activity by BCH resulted in the suppression of cell proliferation in various cancers [9,13,19,20]. Nawashiro *et al.* showed that BCH reduced mortality of C6 glioma-bearing rat model, and suggested that LAT1 inhibitors could be an effective therapeutic option for high-grade gliomas [14]. Kim *et al.* reported that BCH could lead to apoptosis by inducing intracellular depletion of amino acids required for the growth of cancer cells [20]. Liu *et al.* described that BCH induced apoptosis without affecting DNA synthesis in proliferating vascular smooth muscle cells, whereas it had no effect on quiescent smooth muscle cells. Therefore, the inhibition of LAT1 gives rise to growth inhibition effects of highly proliferative cells that require increased amino acid metabolism [36]. Another proposed mechanism of action is cell cycle arrest at G1 phase by the inhibition of LAT1 [37]. However, there is no established explanation regarding the *in vivo* anti-tumor effect of LAT1 inhibitor, although there are two preclinical studies investigating the potential of LAT1 inhibitor in tumor xenografts (glioma [13] and cholangiocarcinoma [current study]). Further *in vivo* study is warranted to evaluate whether a combination of GEM plus LAT1 inhibitor is effective for biliary tract cancer xenograft compared to GEM alone as seen in the current *in vitro* study that has been demonstrating effect of GEM plus BCH.

A recent systemic review has suggested that p53 mutation, cyclins, proliferation indices (Ki-67), mucins, CA19-9, and CEA have potential as prognostic predictors in cholangiocarcinoma [38], however, there is no targeting therapy for these molecules at present. Recently, anti-epidermal growth factor receptor (EGFR) agents, mitogen-activated protein kinase/extracellular-signal regulated kinase (MEK) inhibitors, and anti-angiogenic agents have been thought to be the promising targeted agents for biliary tract cancer [39]. However, the results of clinical trials indicated no therapeutic efficacy to improve the survival of patients with advanced biliary tract cancer [39].

## Conclusion

In conclusion, high expression of LAT1 plays an important role in enhancing tumor growth and cell proliferation and is a promising pathological marker for predicting poor prognosis in patients with biliary tract cancer. The inhibition of LAT significantly suppressed the growth of cholangiocarcinoma, and anti-tumor efficacy of GEM and 5-FU was augmented in combination with LAT inhibitor. Since the LAT1 expression is a significant prognostic marker and LAT1 inhibition probably has anti-tumor efficacy, molecular targeting drug that selectively inhibit LAT1 will aid in the promising therapeutic strategy for bile duct cancer.

## Abbreviations

LAT1: L-type amino acid transporter 1; BCH: 2-aminobicyclo-(2,2,1)-heptane-2-carboxylic acid; MRCP: Magnetic resonance cholangiopancreatography; ERCP: Endoscopic retrograde cholangiopancreatography; EHCC: Extrahepatic cholangiocarcinoma; IHCC: Intrahepatic cholangiocarcinoma; GB: Gallbladder carcinoma; TNM: Pathologic tumor-node-metastasis; MVD: Microvessel density; DMEM: Dulbecco’s modified Eagle’s medium; MTT: 3-[4,5-dimethyl-2-thiazolyl]-2,5-diphenyl-*2H*-tetrazolium bromide; GEM: Gemcitabine; 5-FU: 5-fluorouracil; 18F-FDG: [^18^F]fluoro-2-deoxyglucose; PET: Positron emission tomography; ROI: Region of interest; SUV: Standardized uptake value; OS: Overall survival; PFS: Progression-free survival; MST: Median survival time; EGFR: Epidermal growth factor receptor; MEK: Mitogen-activated protein kinase/extracellular-signal regulated kinase.

## Competing interests

We, all authors, have no financial or personal relationships with other people or organizations that could inappropriately influence our work. The authors declare that they have no competing interests.

## Authors’ contributions

KK, YS, YO, NSI, HT, NO and KA have made substantial contributions to conception and design, or acquisition of data, or analysis and interpretation of data; KK, YO, YK, TO, KS, NS, HI, SN, AY, AS and MI have been involved in drafting the manuscript or revising it critically for important intellectual content; and KK, YO, TO, MM and IT have given final approval of the version to be published. All authors read and approved the final manuscript.

## Pre-publication history

The pre-publication history for this paper can be accessed here:

http://www.biomedcentral.com/1471-2407/13/482/prepub

## Supplementary Material

Additional file 1: Table S1 Primers for realtime RT-PCR used in the present study. Click here for file

Additional file 2: Table S2 Comparison of percentage of high expression and average score of LAT1. Click here for file

Additional file 3: Table S3 Correlation between LAT1 expression and various biomarkers. Click here for file

Additional file 4: Figure S1 Expression of LAT1 and CD98 in human cholangiocarcinoma cell lines (HuCCT1, OZ and HuH28). Representative images from three independent experiments are shown. β-actin was shown as a control. Click here for file
