# Supplementary material for: Clinical significance of L-type amino acid transporter 1 expression as a prognostic marker and potential of new targeting therapy in biliary tract cancer
Source: BMC Cancer. 2013 Oct 16;13:482. doi: 10.1186/1471-2407-13-482 (PMC4016614; doi:10.1186/1471-2407-13-482)
Supplement: Additional file 1: Table S1 — Primers for realtime RT-PCR used in the present study. [file 1471-2407-13-482-S1.doc]

Additional file 1: Table S1. Primers for realtime RT-PCR used in the present study.

|  | Accession No. | Primer sequences | | Expected size (bp) |
| --- | --- | --- | --- | --- |
| LAT1 | AB018009 | sense | ATCGGGAAGGGTGATGTGTCCAAT | 103 |
|  |  | antisense | CAAAGAGGCCGCTGTATAATGCCA |  |
| LAT2 | AB037669 | sense | TTTCCTTCAGGGCTCCTTTGCCTA | 98 |
|  |  | antisense | AGATGGCTCTGGGAAGGTTCTTGT |  |
| LAT3 | AB103033 | sense | TTTGTGACCTTTGTCCTGCACACC | 85 |
|  |  | antisense | TGGATGGGAACACTGCAGCATAGA |  |
| LAT4 | BC027923 | sense | ACCGAGCCAGAGAATGTCACCAAT | 118 |
|  |  | antisense | AATTTAGCATCTCGTCCTGGGCCT |  |
| β-actin | BC002409 | sense | **TCAGAAGGATTCCTATGTGGGCGA** | 109 |
|  |  | antisense | **TTTCTCCATGTCGTCCCAGTTGGT** |  |
