# Supplementary material for: Clinical significance of L-type amino acid transporter 1 expression as a prognostic marker and potential of new targeting therapy in biliary tract cancer
Source: BMC Cancer. 2013 Oct 16;13:482. doi: 10.1186/1471-2407-13-482 (PMC4016614; doi:10.1186/1471-2407-13-482)
Supplement: Additional file 2: Table S2 — Comparison of percentage of high expression and average score of LAT1. [file 1471-2407-13-482-S2.doc]

Additional file 2: Table S2

Comparison of percentage of high expression and average score of LAT1

|  | **Percentage of high expression**  **(%)** | **Average score**  **(mean±SD)** |
| --- | --- | --- |
| **Total patients**  **(n=139)** | 64%  (89/139)  66%  (59/89)  64%  (27/42)  83%  (15/18)  50%  (5/10)  63%  (12/19)  60%  (18/30)  60%  (12/20)  0%  (0/16) | 2.71±0.96    2.76±0.94    2.74±0.88  3.00±0.91  2.50±1.08  2.73±1.04    2.56±1.07    2.70±0.92  1.12±0.34 |
| **Extrahepatic CC**  **(n=89)**    **Lower (n=42)**  **Middle (n=18)**  **Upper (n=10)**  **Hilar (n=19)** |
| **Gallbladder carcinoma**  **(n=30)** |
| **Intrahepatic CC**  **(n=20)** |
| **Control group**  **(n=16)** |

Abbreviation: LAT1. L-type amino acid transporter 1; CC, cholangiocarcinoma.
