# Supplementary material for: Clinical significance of L-type amino acid transporter 1 expression as a prognostic marker and potential of new targeting therapy in biliary tract cancer
Source: BMC Cancer. 2013 Oct 16;13:482. doi: 10.1186/1471-2407-13-482 (PMC4016614; doi:10.1186/1471-2407-13-482)
Supplement: Additional file 3: Table S3 — Correlation between LAT1 expression and various biomarkers. [file 1471-2407-13-482-S3.doc]

Additional file 3: Table S3

Correlation between LAT1 expression and various biomarkers

| **Variable** | | **Spearman γ** | **95% CI** | *p*-value |
| --- | --- | --- | --- | --- |
| **Ki-67** | Total | 0.393 | 0.237-0.529 | **<0.001** |
|  | EHCC | 0.314 | 0.106-0.495 | **0.002** |
|  | IHCC | 0.483 | 0.038-0.768 | **0.031** |
|  | GB | 0.538 | 0.210-0.757 | **0.002** |
| **CD34** | Total | 0.276 | 0.109-0.427 | **0.001** |
|  | EHCC | 0.224 | 0.008-0.416 | **0.036** |
|  | IHCC | -0.001 | -0.109-0.452 | 0.994 |
|  | GB | 0.514 | 0.177-0.742 | **0.003** |

Abbreviation: LAT1, L-type amino acid transporter 1; 95% CI, 95% confidence interval; EHCC, extrahepatic cholangiocarcinoma; IHCC, intrahepatic cholangiocarcinoma; GB, gallbladder carcinoma.
