# Supplementary figures and images for: Clinical significance of L-type amino acid transporter 1 expression as a prognostic marker and potential of new targeting therapy in biliary tract cancer
Source: BMC Cancer. 2013 Oct 16;13:482. doi: 10.1186/1471-2407-13-482 (PMC4016614; doi:10.1186/1471-2407-13-482)

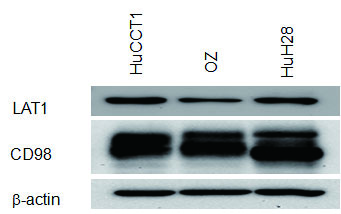

Supplement: Additional file 4: Figure S1 — Expression of LAT1 and CD98 in human cholangiocarcinoma cell lines (HuCCT1, OZ and HuH28). Representative images from three independent experiments are shown. β-actin was shown as a control. [file 1471-2407-13-482-S4.jpg]
